# Molecular Mechanisms of Local Anesthetic Toxicity: From Ion Channel Dysregulation to Mitochondrial Dysfunction and Tissue-Specific Injury

**DOI:** 10.3390/ijms27156900

**Published:** 2026-08-01

**Authors:** Boris Rijavec, Mensur Salihović, Tomislav Mirković

**Affiliations:** 1 Department of Anaesthesiology and Surgical Intensive Care, University Medical Centre Ljubljana, Zaloška cesta 2, SI-1000 Ljubljana, Slovenia; mensur.salihovic@kclj.si (M.S.); tomislav.mirkovic@kclj.si (T.M.); 2 Institute of Pharmacology and Experimental Toxicology, Faculty of Medicine, University of Ljubljana, Korytkova 2, SI-1000 Ljubljana, Slovenia

**Keywords:** local anesthetics, local anesthetic systemic toxicity, tissue-specific toxicity, bupivacaine, lidocaine, mitochondrial dysfunction, ion channel dysregulation, oxidative stress

## Abstract

Local anesthetics are widely used across local and regional anesthesia, pain medicine, emergency care, and dentistry. Their therapeutic effect is based mainly on reversible voltage-gated sodium channel inhibition, but toxicity is not limited to this mechanism. This narrative review integrates molecular evidence on systemic and tissue-specific local anesthetic toxicity. Bupivacaine remains the prototypical cardiotoxic agent, reflecting the convergence of high lipophilicity, persistent cardiac ion channel effects, myocardial accumulation, impaired mitochondrial bioenergetics, altered fatty acid metabolism, calcium dyshomeostasis, and membrane-level injury. Across experimental and translational models, local anesthetics may also induce broad cytotoxicity in various cell types. These effects are primarily concentration- and exposure time-dependent and are modified by route, tissue perfusion, local clearance, metabolic reserve, developmental or physiological vulnerability, and repair capacity. Current experimental, translational, and stem cell-based models provide mechanistic insight but cannot be interpreted as direct clinical dose equivalents. Future studies should link drug type, concentration, formulation, route, exposure duration, patient vulnerability, and tissue-specific outcomes to improve route-specific safety assessment.

## 1. Introduction

Local anesthetics are widely used in regional anesthesia, perioperative analgesia, emergency care, dentistry, pain medicine, dermatologic surgery, and minor surgical procedures because they provide effective and usually reversible sensory blockade. In many settings, they are injected directly into tissue rather than administered only around nerves; examples include local infiltration for skin excision, wound infiltration, field blocks, topical patches, other formulations containing concentrated local anesthetics, and minor soft tissue procedures. This creates high local concentration at the site of administration, making local tissue exposure biologically relevant even when systemic concentrations remain low [1,2,3,4,5].

Bupivacaine is the main example of disproportionate cardiotoxicity. Its toxic profile reflects high lipophilicity, persistent cardiac ion channel effects, myocardial accumulation, negative inotropy, and arrhythmogenic potential. Experimental data also link bupivacaine cardiotoxicity to impaired mitochondrial bioenergetics, altered fatty acid handling, calcium dyshomeostasis, and membrane-level vulnerability [1,6,7,8,9,10].

This review treats local anesthetic toxicity as two related but clinically distinct problems: local anesthetic systemic toxicity (LAST), in which circulating drug affects the central nervous system (CNS) and cardiovascular system, and local or tissue-specific toxicity, in which high local concentration, prolonged exposure, repeated administration, impaired washout, or tissue vulnerability may injure cells near the administration site. Although these clinical settings differ, several molecular injury pathways overlap [1,5,11,12,13].

The aim of this review is not to provide clinical management guidance for LAST or local tissue toxicity. It examines how systemic and local tissue toxicity can be understood at the molecular level, and how local anesthetic exposure may shift from reversible pharmacology to cellular injury [1,5,11,14].

## 2. Scope and Conceptual Framework of Review

This narrative review focuses on molecular mechanisms shared by local anesthetic systemic toxicity and direct tissue-specific injury. These topics are often considered separately: systemic toxicity is usually discussed through central nervous system and cardiovascular manifestations, whereas local toxicity is usually framed as injury at or near the site of administration. This separation is clinically useful, but biologically incomplete. In both settings, toxicity reflects the interaction between physicochemical drug properties, concentration–time exposure, tissue distribution, local or systemic clearance, and cellular vulnerability.

The review begins with physicochemical determinants of toxicity and receptor or ion channel-level effects. It then examines mitochondrial bioenergetic failure, altered fatty acid metabolism, calcium dyshomeostasis, and membrane vulnerability. Subsequent sections address cellular stress and regulated injury pathways, including endoplasmic/sarcoplasmic reticulum stress, apoptosis, dysregulated autophagy or mitophagy, ferroptosis, senescence, and impaired repair. Organ- and tissue-specific injury patterns are then summarized, followed by a discussion of human induced pluripotent stem cell-derived cardiomyocyte models and in silico toxicology as tools for mechanistic interpretation and future risk prediction [8,9,10,11,15,16,17].

### Literature Search and Selection Strategy

Targeted literature searches were conducted in PubMed/MEDLINE and Web of Science, with additional Scopus tracking and manual screening of reference lists. Publications from January 2019 to May 2026 were prioritized. Older studies were retained when they provided foundational mechanistic evidence. Search terms combined local anesthetic names with toxicity- and mechanism-related terms, including cardiotoxicity, neurotoxicity, myotoxicity, chondrotoxicity, mitochondria, oxidative phosphorylation, reactive oxygen species (ROS), calcium homeostasis, endoplasmic/sarcoplasmic reticulum (ER/SR) stress, autophagy, mitophagy, ferroptosis, cardiolipin, carnitine, fatty acid oxidation, lipid emulsion, and tissue injury. During revision, targeted supplementary searches were also performed for lipid emulsion rescue mechanisms, local anesthetic adjuvants, metabolomics, multi-omics, and systems toxicology.

**Figure 1:**
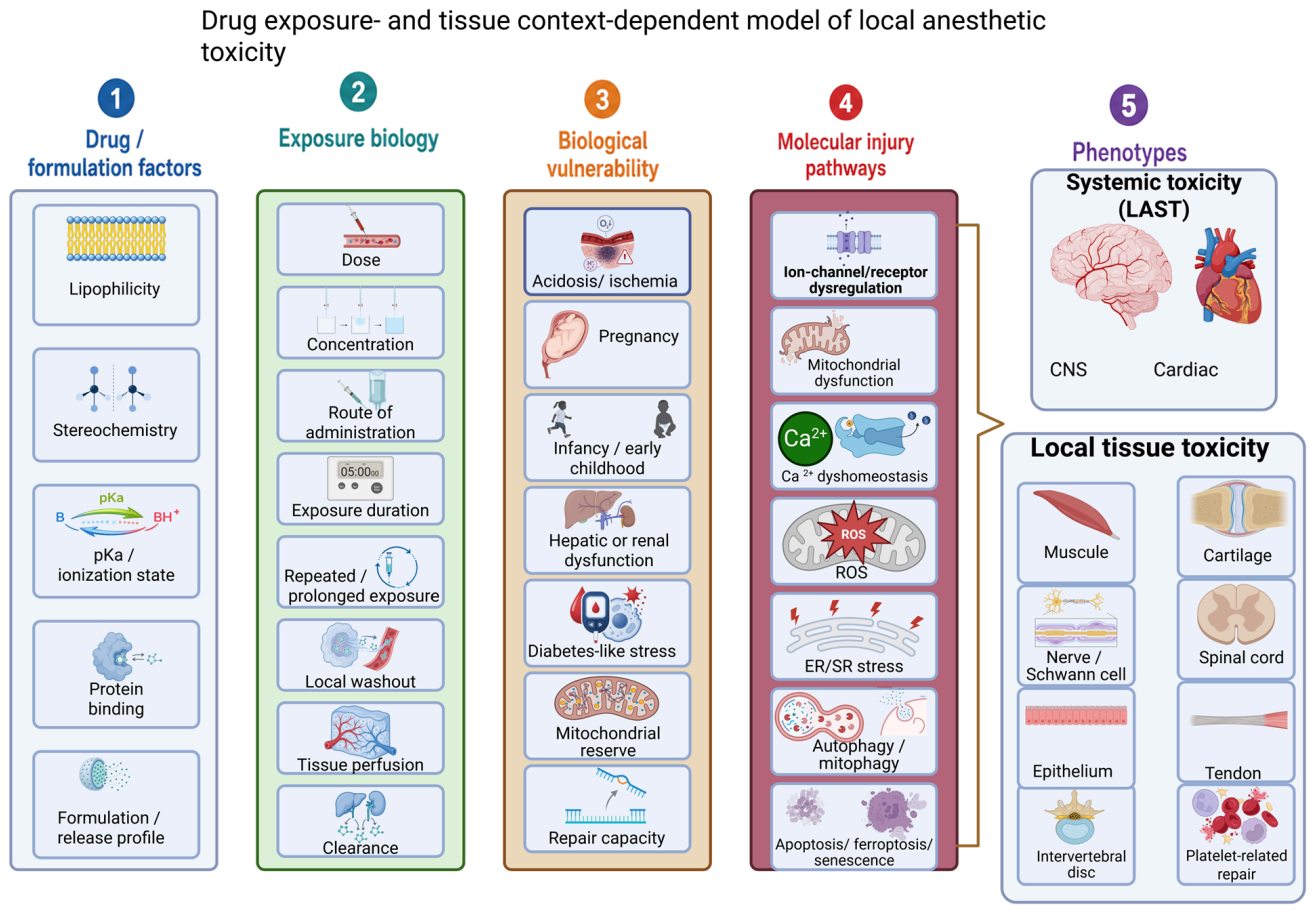
Drug exposure- and tissue context-dependent model of local anesthetic toxicity. The figure illustrates the conceptual framework of this review. Local anesthetic toxicity is shaped by the interaction of drug/formulation factors, exposure biology, biological vulnerability, and molecular injury pathways, which together determine the final phenotype. Depending on concentration, duration, route, local washout, tissue perfusion, clearance, and host susceptibility, toxicity may manifest as systemic toxicity (LAST; CNS and cardiac) or as tissue-specific injury affecting muscle, cartilage, peripheral nerve/Schwann cells, spinal cord, epithelium, tendon, intervertebral disk, and platelet-related repair systems. Major molecular pathways include ion channel/receptor dysregulation, mitochondrial dysfunction, Ca^2+^ dyshomeostasis, ROS generation, ER/SR stress, dysregulated autophagy/mitophagy, and regulated cell death. Abbreviations: LAST, local anesthetic systemic toxicity; CNS, central nervous system; pKa, acid dissociation constant; Ca^2+^, calcium ion; ROS, reactive oxygen species; ER/SR, endoplasmic/sarcoplasmic reticulum. Created in BioRender. Rijavec, B. (2026) https://BioRender.com/t4groig (accessed on 28 July 2026).

After initial deduplication, PubMed/MEDLINE yielded 921 records. Exclusion of retractions and expressions of concern left 912 articles for mechanistic screening. Web of Science added 292 unique records, while Scopus was used for gap checking and citation tracking. Original in vitro and in vivo experimental studies, translational studies, and authoritative reviews were prioritized. Because this article is a mechanistic narrative review rather than a systematic review or meta-analysis, no formal risk-of-bias assessment, quantitative evidence synthesis, or Preferred Reporting Items for Systematic Reviews and Meta-Analyses (PRISMA) flow diagram was prepared. The selected evidence was organized around drug and formulation factors, exposure biology, biological vulnerability, molecular injury pathways, and systemic or tissue-specific phenotypes, as summarized in Figure 1.

## 3. Chemical and Pharmacological Determinants of Local Anesthetic Toxicity

The toxic potential of local anesthetics arises from the same chemical features that make them clinically useful. Their common structure includes a lipophilic aromatic group, an ester or amide linkage, and a hydrophilic amine. This arrangement allows for membrane passage and sodium channel inhibition. It also permits interaction with lipid membranes, intracellular organelles, non-sodium ion channels, receptors, selected enzymes, and mitochondrial systems [1,14].

Lipophilicity is a major determinant of both potency and toxicity. Greater lipid solubility increases membrane penetration, protein binding, duration of action, tissue retention, and access to lipid-rich intracellular compartments. Bupivacaine illustrates this relationship. Its high lipophilicity contributes to myocardial accumulation, persistent interaction with cardiac sodium channels, mitochondrial disturbance, and disproportionate cardiotoxicity. Ropivacaine is less lipophilic, which is one reason for its more favorable systemic safety profile among long-acting amide local anesthetics [2,7,15].

Lipophilicity also matters for direct cellular injury. In human T-lymphoma models, local anesthetic cytotoxicity correlated more closely with octanol/buffer partition coefficients and clinical potency than with ester/amide linkage or stereochemistry. This supports the importance of membrane access and intracellular distribution in direct cytotoxicity. S-enantiomer formulations reduce some systemic risks, especially cardiac conduction-related risk, but they do not abolish local tissue or cellular injury during prolonged or high-concentration exposure [3,18].

Ionization and local pH modify these effects. Local anesthetics are weak bases that shift between uncharged and protonated forms. The uncharged fraction favors lipid membrane passage. The protonated form contributes to sodium channel binding and electrostatic interaction with anionic membrane lipids. Acidic or ischemia-like conditions may therefore increase membrane-associated toxicity by altering sodium channel behavior and strengthening interactions with anionic membrane systems [1,10,14,19].

Protein binding and tissue accumulation influence systemic toxicity. Highly protein-bound agents may have low free plasma concentrations under stable conditions, yet still accumulate in well-perfused or lipid-rich organs such as the heart and brain. In isolated guinea pig hearts, myocardial concentrations of bupivacaine and ropivacaine exceeded arterial perfusate concentrations. These changes were accompanied by conduction slowing, reduced left ventricular pressure, impaired contractility, and reversible mitochondrial swelling [1,6,15].

Stereochemistry modifies toxicity among long-acting amide local anesthetics. Racemic bupivacaine contains both R- and S-enantiomers, whereas levobupivacaine and ropivacaine are pure S-enantiomer formulations developed to improve systemic safety. Their lower propensity for severe cardiac conduction disturbances and malignant arrhythmias supports the toxicological relevance of stereochemistry. Lipophilicity, myocardial accumulation, mitochondrial effects, calcium dyshomeostasis, and membrane interactions remain important drivers of myocardial and cellular injury [2,7,18].

The ester or amide linkage affects metabolism, stability, allergic potential, and duration of action. It is less predictive of direct cellular toxicity than exposure conditions. Concentration, exposure duration, potency, lipophilicity, local clearance, and tissue vulnerability appear more relevant when local exposure becomes cytotoxic. During repeated, prolonged, or catheter-based administration, cumulative dose and pharmacokinetics also become important, so favorable single-dose pharmacokinetics should not be interpreted as absence of exposure-related risk [1,3,14,20].

Concentration and exposure time are central to local toxicity. Under prolonged, repeated, or high-concentration conditions, local anesthetics can affect chondrocytes, keratinocytes, skeletal muscle cells, Schwann cells, corneal epithelial cells, neuronal cells, tendon cells, intervertebral disk cells, and other tissue-specific models. The threshold for injury may be lowered by inflammation, ischemia-like conditions, mitochondrial vulnerability, oxidative stress, diabetes-like metabolic stress, impaired clearance, or limited repair capacity [4,5,11,12,13,19,21,22,23].

Local anesthetic toxicity is best understood as an exposure-dependent interaction between drug chemistry and biological context. Drug identity matters, but it does not act in isolation. Local anesthetic concentration, exposure duration, route of administration, tissue perfusion, protein binding, tissue accumulation, local clearance, and cellular vulnerability determine whether exposure produces reversible pharmacology or progresses toward mitochondrial injury, oxidative stress, ER/SR stress, regulated cell death, and tissue-specific damage [1,3,5,11,13,14,19].

## 4. Ion Channel and Receptor-Level Mechanisms

The therapeutic action of local anesthetics is usually described through reversible inhibition of voltage-gated sodium channels. At toxic concentrations, this explanation is too narrow. Local anesthetics can also affect other ion channels and receptor-operated conductances in neurons, cardiomyocytes, peripheral nerves, and skeletal muscle. These effects may contribute to seizures, conduction delay, arrhythmogenesis, myocardial depression, altered calcium handling, and tissue-specific injury. They also provide an early functional link to later mitochondrial dysfunction, oxidative stress, ER/SR stress, and regulated cell death [1,2,14].

### 4.1. Voltage-Gated Sodium Channels and State-Dependent Block

Local anesthetics preferentially interact with activated or inactivated sodium channel states. This state-dependent block explains part of their therapeutic selectivity, but it also becomes toxic in highly excitable tissues, especially myocardium and the central nervous system. In cardiomyocytes, sodium channel blockade slows phase 0 depolarization, reduces conduction velocity, and prolongs PR/QRS intervals. These changes create a substrate for conduction delay, re-entry, and malignant arrhythmias. Bupivacaine has greater arrhythmogenic potential because it dissociates slowly from cardiac sodium channels, producing more persistent conduction disturbance than less lipophilic agents such as lidocaine or ropivacaine [1,2,7,14,24].

Human cardiac voltage-gated sodium channel isoform 1.5 (NaV1.5) studies support this mechanism. Lidocaine and levobupivacaine inhibit adult and neonatal NaV1.5 channels in an inactivation-dependent manner. Tetrodotoxin-sensitive sodium channel α-subunits also contribute to local anesthetic inhibition of cardiac sodium currents. These findings confirm sodium channel block as a crucial component of cardiotoxicity. They also show its limits: severe cardiotoxicity cannot be explained by isolated NaV1.5 inhibition alone [7,25,26].

Lipid emulsion experiments make the same point. Part of the rescue effect may involve reduced bupivacaine inhibition of NaV1.5, but this is only one mechanism. Drug redistribution, fatty acid substrate support, calcium handling, mitochondrial function, and reversal of carnitine–acylcarnitine translocase inhibition also contribute in experimental models. Severe bupivacaine cardiotoxicity is therefore better understood as a combined electrophysiological, mitochondrial, metabolic, and membrane-related injury rather than as sodium channel block alone [27,28,29].

### 4.2. Potassium Channels and Metabolic–Electrical Coupling

Potassium channels are another target of local anesthetics. Their inhibition can affect repolarization, resting membrane potential, neuronal firing, and metabolic–electrical coupling. In neuronal models, bupivacaine inhibits human neuronal voltage-gated potassium 3 (Kv3) channels, which may impair rapid repolarization and high-frequency firing. Deletion of TWIK-related acid-sensitive potassium (TASK) channels reduces sensitivity to local anesthetic-induced seizures. These findings suggest that potassium channel effects contribute to CNS excitability, although they do not replace GABAergic, glutamatergic, or sodium channel mechanisms [30,31].

TWIK-related potassium channel 1 (TREK-1) connects membrane excitability with mitochondrial function and cell survival. Bupivacaine, levobupivacaine, and ropivacaine inhibit TREK-1 with different potencies. In cardiomyoblast models, TREK-1 has been linked to bupivacaine-induced changes in cell viability, membrane potential, mitochondrial membrane potential, intracellular calcium, and lipid emulsion responsiveness [32,33].

Cardiac potassium channels add a metabolic component to electrical toxicity. Sarcolemmal adenosine triphosphate-sensitive potassium (KATP) channels couple cellular energy status to membrane excitability and help protect the myocardium during ischemic or metabolic stress. Bupivacaine, levobupivacaine, and ropivacaine inhibit these conductances, which may weaken this adaptive response [34].

Small-conductance calcium-activated potassium 2 (SK2) channels are also relevant in bupivacaine cardiotoxicity. Bupivacaine directly inhibits SK2 currents. In isolated-heart and in vivo mouse models, SK2 ablation or deletion delayed arrhythmogenesis, asystole, QT prolongation, and heart-rate decline. These data support a contribution of calcium-activated potassium currents to bupivacaine-induced cardiac electrical instability [35,36,37].

### 4.3. Calcium Dyshomeostasis and Excitation–Contraction Failure

Calcium dyshomeostasis links ion channel toxicity with mitochondrial injury, contractile failure, and arrhythmogenesis. Bupivacaine inhibits L-type calcium currents in ventricular cardiomyocytes by altering channel availability and shifting channels toward inactivated states. In neonatal rat ventricular models, bupivacaine also suppresses voltage-gated calcium channel subtype 1.3 (CaV1.3)-related calcium current signaling through a calreticulin-associated mechanism. These effects can disturb excitation–contraction coupling and add to sodium channel-mediated cardiotoxicity [38,39].

Human induced pluripotent stem cell-derived cardiomyocyte models show the same vulnerability at the cellular level. Bupivacaine disrupts intracellular calcium dynamics more strongly than ropivacaine, with reduced calcium wave amplitude and altered contraction–relaxation kinetics. Calcium supplementation partly mitigates bupivacaine-induced contractile and rhythm disturbances, whereas nifedipine worsens toxicity in this model. These findings are experimental and should not be translated directly into clinical calcium treatment. They support a narrower conclusion: disturbed calcium flux and impaired excitation–contraction coupling contribute to the heightened cardiotoxic profile of bupivacaine under susceptible conditions [40].

### 4.4. GABAergic, Glutamatergic, and Hyperpolarization-Activated Cyclic Nucleotide-Gated (HCN)-Mediated CNS Excitability

Local anesthetic-induced CNS toxicity often follows a biphasic pattern. Early toxicity is dominated by excitation and seizures; higher concentrations may then produce dose-dependent CNS depression. The excitatory phase is largely explained by loss of inhibitory tone. Experimental studies show that local anesthetics can suppress presynaptic γ-aminobutyric acid (GABA) release, reduce γ-aminobutyric acid type A (GABA-A) receptor-mediated currents in a subunit-dependent manner, and, particularly with lidocaine, shift the GABA-induced reversal potential in a depolarizing direction. These effects weaken chloride-dependent inhibition and may lower the seizure threshold [41,42,43,44].

Glutamatergic signaling adds to this excitatory state. Antagonist studies involving N-methyl-D-aspartate (NMDA) and non-NMDA receptors support glutamate-system involvement in lidocaine- and bupivacaine-induced seizures. High-concentration intrathecal exposure can also provoke dose-dependent glutamate release and neuronal injury. Hyperpolarization-activated cyclic nucleotide-gated (HCN) channels form a separate modulatory target. Lidocaine inhibits HCN-mediated hyperpolarization-activated currents (Ih) in neuronal and heterologous models. These effects are best interpreted as modifiers of neuronal excitability, rebound depolarization, rhythmicity, and nociceptive signaling, rather than as primary seizure triggers [45,46,47,48,49].

Muscarinic receptor signaling represents an additional receptor-level target of local anesthetics, particularly for lidocaine. Experimental studies in bovine tracheal smooth muscle suggest that lidocaine can interfere with muscarinic receptor-mediated signaling, including muscarinic acetylcholine receptor subtype 2 (M2 receptor)-dependent inhibition of relaxation pathways and muscarinic inhibition of adenylyl cyclase. This may be relevant to airway smooth muscle tone and pulmonary receptor pharmacology, but it should be interpreted as an adjunct receptor-operated mechanism rather than a primary explanation for LAST. In the heart, M2 receptors are central to vagal regulation of heart rate and atrioventricular conduction; however, lidocaine-related cardiac toxicity and antiarrhythmic effects remain primarily explained by state-dependent sodium channel blockade, with muscarinic modulation representing a possible secondary contributor to autonomic or receptor-level effects rather than the dominant mechanism [50,51].

### 4.5. Integrated Interpretation

Ion channel and receptor effects explain the early functional toxicity of local anesthetics. Sodium channel block slows conduction. Potassium-channel effects alter repolarization and neuronal firing. Calcium dysregulation impairs excitation–contraction coupling. GABAergic inhibition is weakened, while glutamatergic signaling can promote seizure propagation. HCN channel effects further modify excitability and nociceptive processing. These early functional disturbances are important because they can precede, and partly drive, mitochondrial dysfunction, oxidative stress, ER/SR stress, and regulated cell death [1,11,14,25,31,40,52].

Muscarinic receptor signaling, particularly M2-related airway and autonomic pathways described for lidocaine, may further modify receptor-operated responses in selected tissues, but it should be interpreted as an adjunct mechanism rather than a dominant explanation for local anesthetic systemic toxicity or cardiotoxicity [50,51].

## 5. CNS Toxicity: From Network Disinhibition to Neuronal Injury

The central nervous system is one of the earliest clinical targets of local anesthetic systemic toxicity. Initial toxicity often presents with prodromal excitation, agitation, sensory disturbance, or seizures. With higher concentrations, this may progress to dose-dependent CNS depression. This biphasic pattern reflects a shift from network disinhibition to broader suppression of neuronal activity [1,2].

At the molecular level, CNS toxicity involves more than sodium channel block. Local anesthetics can alter voltage-gated sodium channels, background potassium conductances, GABAergic inhibition, glutamatergic excitation, chloride homeostasis, calcium influx, and neuronal bioenergetics. When exposure is brief, these effects may remain functional and reversible. With higher, prolonged, neuraxial, or metabolically amplified exposure, the same disturbance may exceed cellular reserve and trigger mitochondrial dysfunction, oxidative stress, endoplasmic reticulum stress, and regulated cell death [1,31,42,44,53].

### 5.1. GABAergic Disinhibition, Chloride Homeostasis, and Seizure Generation

The early seizure phenotype is mainly explained by loss of inhibitory control with relative excitatory predominance. Experimental studies show reduced presynaptic GABA release, inhibition of GABA-A receptor-mediated currents, subunit-dependent receptor effects, and lidocaine-related depolarizing shift of the GABA-induced reversal potential. These findings support a mechanism in which weakened chloride-dependent inhibition lowers the seizure threshold [41,42,43,44].

Glutamatergic signaling contributes to seizure propagation and excitotoxic injury. Experimental antagonist studies implicate N-methyl-D-aspartate (NMDA) and non-NMDA receptors in lidocaine- and bupivacaine-induced seizures. High-concentration intrathecal exposure can also produce dose-dependent glutamate release and neuronal injury [45,46,47].

Local anesthetic effects on excitatory neurotransmission may also involve NMDA receptor signaling. Human recombinant NMDA receptor studies show that several local anesthetics can inhibit glutamate/glycine-induced receptor activation, while spinal models suggest that high intrathecal concentrations may increase glutamate release and contribute to neuronal injury. These findings support the view that CNS toxicity is not only a consequence of sodium channel blockade or loss of inhibitory tone, but also reflects disturbed excitatory–inhibitory receptor signaling under high-concentration or neuraxial exposure conditions [47,54,55].

### 5.2. Mitochondrial Dysfunction, Oxidative Stress, and ER Stress in Neuronal Injury

Mitochondria are central to local anesthetic-induced neuronal injury. Neurons require mitochondrial oxidative phosphorylation for adenosine triphosphate (ATP) production, calcium buffering, redox control, and synaptic function. Local anesthetics can impair mitochondrial membrane potential, disturb respiratory chain function, increase reactive oxygen species generation, and activate intrinsic apoptotic pathways. Lidocaine-induced neuronal injury is linked to mitochondrial damage and caspase activation. In SH-SY5Y human neuroblastoma cells, bupivacaine induces apoptosis through reactive oxygen species production, complex I/III impairment, loss of membrane potential, endoplasmic reticulum stress, and caspase activation [56,57].

Ropivacaine neurotoxicity also involves mitochondrial dynamics. In SH-SY5Y cells, ropivacaine induces fission-like mitochondrial changes, increases dynamin-related protein 1 expression, reduces mitochondrial membrane potential, impairs cytochrome c oxidase activity and ATP production, and increases reactive oxygen species generation. Silencing dynamin-related protein 1 attenuates these changes. Recent experimental data also suggest that methyltransferase-like 3 (METTL3)-mediated regulation of brain-derived neurotrophic factor (BDNF) may modify bupivacaine-induced oxidative and mitochondrial damage [58,59].

Endoplasmic reticulum stress provides another route from local anesthetic exposure to neuronal apoptosis. Lidocaine induces ER stress-associated apoptosis in vitro and in vivo. In rat pheochromocytoma PC12 cells, this includes upregulation of ER chaperones and stress sensors, including binding immunoglobulin protein (BiP), calnexin, calreticulin, protein disulfide isomerase, activating transcription factor 6 (ATF6), inositol-requiring enzyme 1 (IRE1), and protein kinase R-like endoplasmic reticulum kinase (PERK), together with eukaryotic initiation factor 2 alpha (eIF2α) phosphorylation and X-box binding protein 1 (*XBP1*) messenger RNA (mRNA) splicing. Lidocaine also modifies B-cell lymphoma 2 (Bcl-2) family signaling, downregulating Bcl-2 and B-cell lymphoma-extra large (Bcl-xL) while increasing pro-apoptotic Bcl-2 homologous antagonist/killer (Bak) and Bcl-2-associated X protein (Bax) expression. Bupivacaine spinal neurotoxicity is also linked to ER stress pathways, and high-glucose conditions amplify bupivacaine-induced apoptosis through combined mitochondrial dysfunction and ER stress [52,60,61,62].

### 5.3. Autophagy, Mitophagy, Ferroptosis, and Metabolic Vulnerability

Autophagy and mitophagy are protective quality control pathways, but they may become maladaptive when cellular stress is excessive or autophagic flux is impaired. In high-glucose models, bupivacaine-induced neuronal injury is amplified through apoptosis, mitochondrial dysfunction, endoplasmic reticulum stress, and disrupted autophagy. Reported pathways include PERK–activating transcription factor 4 (ATF4)–C/EBP homologous protein (CHOP), IRE1–tumor necrosis factor receptor-associated factor 2 (TRAF2), and reactive oxygen species-dependent taurine-upregulated gene 1 (*TUG1*)/mechanistic target of rapamycin (mTOR) signaling in dorsal root ganglion (DRG) neurons. Lidocaine neurotoxicity in diabetic Goto-Kakizaki rats has been linked to AMP-activated protein kinase (AMPK)-mediated mitophagy, whereas ropivacaine-induced neuronal apoptosis involves excessive PTEN-induced kinase 1 (PINK1)/Parkin-mediated mitophagy [62,63,64,65,66].

A pregnant mouse spinal model adds another vulnerability context. In that model, bupivacaine-induced neurotoxicity involved oxidative DNA damage, poly(ADP-ribose) polymerase 1 (PARP-1) activation, and autophagic flux disruption [67]. Ferroptosis has also been implicated in experimental spinal neurotoxicity. Ferrostatin-1 attenuated bupivacaine-induced injury in rats by reducing lipid peroxidation and iron accumulation while preserving glutathione peroxidase 4 and cystine/glutamate antiporter (xCT) defenses [68]. Lidocaine models further suggest ferritinophagy-mediated ferroptosis, involving nuclear receptor coactivator 4 (NCOA4), CDGSH iron-sulfur domain-containing protein 2 (CISD2), glutathione peroxidase 4 (GPX4), and solute carrier family 7 member 11 (SLC7A11) [69]. These data remain model-specific. Ferroptosis should therefore be presented as an emerging pathway, not as an established universal mechanism of local anesthetic neurotoxicity.

### 5.4. Spinal, Peripheral Glial, and Developmental Vulnerability

Spinal and intrathecal exposure can produce high local concentrations near neural tissue. This setting may reveal mechanisms that are not captured by systemic plasma toxicity. Lidocaine has long been associated with transient neurologic symptoms and concern for spinal neurotoxicity. Proposed mechanisms include direct neuronal injury, calcium dyshomeostasis, mitochondrial dysfunction, and concentration-dependent cellular damage. Intrathecal levobupivacaine also shows concentration-dependent neurotoxicity in rat spinal models. Repeated intrathecal lidocaine exposure increases paw-withdrawal thresholds, causes spinal histopathological injury, and alters long non-coding RNA (lncRNA) and messenger RNA (mRNA) expression, with enrichment of cell cycle and immune-inflammatory pathways [70,71,72].

Local anesthetic neurotoxicity is not limited to neurons. In Schwann cells, articaine and lidocaine trigger toxicity through disrupted metabolism, impaired mitochondrial bioenergetics, endoplasmic reticulum stress, and apoptosis. Broader glial responses may be protective or toxic depending on anesthetic type, concentration, exposure duration, and pathological context. Developing neural cells may be especially sensitive. In experimental models, ropivacaine impairs mitochondrial function and calcium oscillations in oligodendrocyte precursor cells (OPCs), leading to developmental spinal dysmyelination through protein kinase B (Akt) signaling inhibition [21,73,74].

### 5.5. Integrated Interpretation

CNS toxicity can be read as a progression from network dysfunction to cellular injury. Early systemic toxicity is mainly functional: inhibitory control weakens, chloride homeostasis shifts, potassium-channel modulation changes excitability, and glutamatergic signaling can promote seizure propagation. Brief exposure may remain reversible. Higher local concentration, neuraxial exposure, prolonged contact, or metabolic vulnerability can shift the same process toward mitochondrial injury, oxidative stress, ER stress, impaired autophagy or mitophagy, ferroptosis-related pathways, and glial or developmental injury [1,21,31,41,44,52,57,66,68,74].

## 6. Mitochondrial, Metabolic, and Membrane Toxicity

Mitochondria are a major target in severe local anesthetic cardiotoxicity, especially with long-acting lipophilic amides such as bupivacaine. Cardiac sodium channel blockade remains important, but it does not explain the full phenotype. Severe cardiotoxicity also involves calcium dyshomeostasis, impaired mitochondrial bioenergetics, altered fatty acid metabolism, oxidative stress, and membrane biophysical injury [1,2,7,40].

### 6.1. Mitochondrial Bioenergetic Failure in Cardiotoxicity

The heart depends heavily on mitochondrial oxidative phosphorylation for ATP production. When local anesthetics impair mitochondrial function, contractility, calcium handling, membrane stability, and electrical conduction may deteriorate rapidly. Experimental studies show that amide local anesthetics, including lidocaine and bupivacaine, can acutely inhibit myocardial oxygen consumption and reduce ATP availability. In murine ventricular tissue, bupivacaine produces stronger respiratory inhibition than lidocaine. Other mitochondrial preparations show disruption of oxidative phosphorylation, mitochondrial membrane potential, respiratory chain function, and membrane permeability, depending on concentration, substrate conditions, and mitochondrial access [8,15,75,76].

Bupivacaine remains the main example of this toxicity because high lipophilicity, persistent cardiac sodium channel effects, and mitochondrial impairment occur together. Comparative work shows that both bupivacaine and ropivacaine can disturb mitochondrial function, but bupivacaine more strongly inhibits oxygen consumption and ATP-linked bioenergetics. Ropivacaine is generally less disruptive, although experimental mitochondrial toxicity can appear rapidly when membrane access is facilitated [2,7,8,76].

Myocardial accumulation connects pharmacokinetics with mitochondrial injury. In isolated guinea pig hearts, bupivacaine and ropivacaine accumulated in myocardial tissue at concentrations higher than those measured in arterial perfusate. This was accompanied by PR and QRS prolongation, reduced left ventricular pressure, impaired contractility, and reversible mitochondrial swelling. Mitochondrial changes reversed more rapidly after ropivacaine than after bupivacaine, consistent with lower tissue persistence and a more favorable systemic safety profile [15].

### 6.2. Calcium Dyshomeostasis, Oxidative Stress, and Self-Amplifying Cardiotoxicity

Mitochondrial dysfunction and calcium dyshomeostasis reinforce each other during cardiotoxicity. ATP depletion can impair sodium–potassium adenosine triphosphatase (Na^+^/K^+^-ATPase) activity, sarcoplasmic reticulum calcium cycling, plasma membrane calcium extrusion, and mitochondrial membrane potential. Calcium overload can then promote mitochondrial calcium uptake, permeability transition, respiratory chain dysfunction, and reactive oxygen species generation. This links ion channel disturbance with mitochondrial injury, oxidative stress, and contractile failure [8,28,40].

Severe local anesthetic cardiotoxicity often unfolds in a worsening physiological environment. Hypoxia, ischemia, reduced perfusion, and acidosis can modify sodium channel gating, increase the protonated fraction of the local anesthetic, alter tissue distribution, and strengthen interactions with anionic membrane lipids. Once conduction slowing and myocardial depression reduce cardiac output, hypoxia and acidosis may worsen further. The result is a toxic cycle involving electrophysiological instability, mitochondrial dysfunction, reactive oxygen species generation, calcium dyshomeostasis, permeability transition susceptibility, and ATP depletion [1,15,19,40].

### 6.3. Carnitine Shuttle, Fatty Acid Oxidation, and Metabolic Vulnerability

Bupivacaine cardiotoxicity also involves myocardial fuel handling. Adult cardiomyocytes depend strongly on mitochondrial oxidative phosphorylation and fatty acid oxidation for ATP production. Bupivacaine inhibits acylcarnitine exchange in cardiac mitochondria, especially when respiration depends on acylcarnitine substrates. This impairs lipid-supported mitochondrial respiration and adds to the bioenergetic deficit described in other cardiotoxicity models [9,28,29].

This mechanism helps link fatty acid transport with the bupivacaine phenotype. Reduced fatty acid entry into the mitochondrial matrix may compromise ATP production in high-demand tissues. ATP loss can then impair Na^+^/K^+^-ATPase activity, calcium cycling, sarcoplasmic reticulum function, myofilament contraction, and membrane stability. In experimental models, carnitine deficiency increases susceptibility to bupivacaine cardiotoxicity, whereas prophylactic L-carnitine reduces it. Once bupivacaine-induced cardiac arrest is established, L-carnitine alone does not restore spontaneous circulation. Carnitine biology should therefore be presented as a metabolic vulnerability and possible preventive experimental target, not as a rescue treatment for severe local anesthetic systemic toxicity [9,77,78].

### 6.4. Membrane Perturbation and Cardiolipin-Related Mitochondrial Vulnerability

Local anesthetics can also interact directly with lipid membranes. Their amphiphilic structure allows for insertion into lipid interfaces, where they may alter membrane packing and bilayer fluidity. In Langmuir monolayer studies, amide local anesthetics produced monolayer expansion and fluidization, with stronger effects in cardiolipin-containing systems and modulation by cholesterol content. Membrane composition therefore influences local anesthetic–membrane interaction [10,79].

Cardiolipin is concentrated in the inner mitochondrial membrane. It supports respiratory chain organization, oxidative phosphorylation, membrane architecture, mitochondrial dynamics, mitophagy, cytochrome c binding, apoptosis, and permeability transition signaling. Altered cardiolipin content, oxidation state, acyl-chain composition, or protein binding can impair electron transport, increase reactive oxygen species generation, destabilize respiratory supercomplexes, facilitate cytochrome c release, and lower the threshold for mitochondrial permeability transition [80,81].

Local anesthetic interaction with cardiolipin-containing membranes may therefore contribute to mitochondrial and cardiac toxicity. The evidence is mainly experimental. Bupivacaine disrupts cardiolipin-rich biomimetic liposomes. Dibucaine shows cardiolipin- and phosphatidylethanolamine-dependent partitioning, fluidization, and alteration of mitochondrial membrane models. These findings support membrane-level vulnerability, but they do not establish cardiolipin as the main clinical target of local anesthetic cardiotoxicity. The better interpretation is that membrane effects add to ion channel disruption, calcium dyshomeostasis, impaired fatty acid oxidation, and mitochondrial bioenergetic failure [79,82,83,84].

### 6.5. Integrated Interpretation

Lipid emulsion rescue is relevant here only as mechanistic support, not as a treatment focus. Experimental data suggest that recovery from severe bupivacaine cardiotoxicity may involve drug redistribution, partial reversal of NaV1.5 inhibition, fatty acid substrate support, attenuation of carnitine–acylcarnitine translocase inhibition, improved calcium handling, and mitochondrial protection. This reinforces the interpretation that severe cardiotoxicity reflects combined electrophysiological, metabolic, mitochondrial, and membrane-level injury rather than sodium channel blockade alone [27,28,29,75,77].

**Figure 2:**
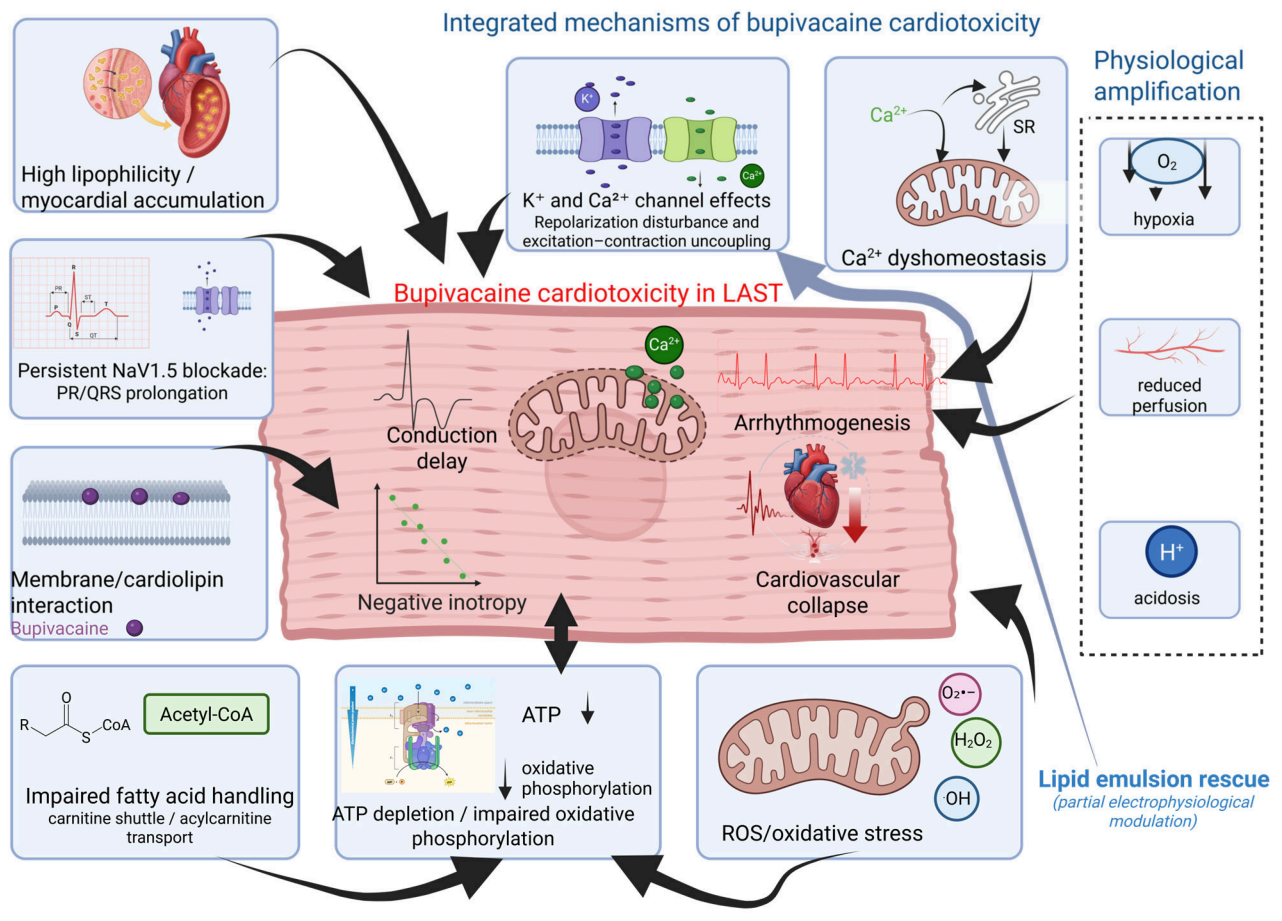
Integrated mechanisms of bupivacaine cardiotoxicity in LAST. Bupivacaine cardiotoxicity reflects the convergence of high lipophilicity, myocardial accumulation, persistent NaV1.5 blockade, potassium and calcium channel effects, mitochondrial bioenergetic failure, impaired fatty acid handling, calcium dyshomeostasis, oxidative stress, membrane/cardiolipin interactions, and physiological amplification by hypoxia, ischemia, acidosis, and reduced perfusion. Lipid emulsion rescue is shown as a multi-component intervention involving dynamic lipid shuttle/drug redistribution, fatty acid substrate support, partial electrophysiological modulation, nitric oxide and kinase pathway modulation, and mitochondrial support. Black arrows indicate contributory and self-amplifying toxic pathways, whereas the blue arrow indicates the partial electrophysiological modulation component of lipid emulsion rescue. Created in BioRender. Rijavec, B. (2026) https://BioRender.com/mgk54fk (accessed on 28 July 2026).

At the molecular level, lipid emulsion therapy should not be interpreted as a single-mechanism antidote. Contemporary mechanistic reviews support a combined model in which lipid emulsion acts through both indirect drug redistribution and direct cellular effects. The indirect component is commonly described as a dynamic lipid shuttle, in which lipophilic local anesthetics, particularly bupivacaine, are sequestered from highly perfused target organs such as the heart and brain and redistributed toward compartments involved in storage, metabolism, and detoxification. Direct effects may include provision of fatty acid substrate for mitochondrial energy metabolism, partial attenuation of mitochondrial dysfunction, positive inotropic effects, modulation of nitric oxide signaling, activation of prosurvival Akt/glycogen synthase kinase-3 beta (GSK-3β) pathways and modulation of the mitochondrial permeability transition pore, and partial reversal or modulation of cardiac sodium channel blockade. Thus, lipid emulsion rescue pharmacology intersects with the same electrophysiological, mitochondrial, metabolic, and membrane-level injury pathways that underlie severe bupivacaine cardiotoxicity [85,86]. These integrated mechanisms are summarized in Figure 2.

## 7. ROS, Calcium Dyshomeostasis, ER/SR Stress, Autophagy, and Regulated Cell Death

Cellular stress pathways help explain how local anesthetic exposure can progress from reversible pharmacology to tissue injury. Receptor and ion channel effects are often early events, but they do not fully explain persistent cytotoxicity. In neural, cardiac, skeletal muscle, cartilage, glial, epithelial, and connective tissue models, local anesthetic exposure has been linked to reactive oxygen species generation, calcium dyshomeostasis, mitochondrial membrane-potential loss, endoplasmic/sarcoplasmic reticulum stress, impaired autophagy or mitophagy, apoptosis, ferroptosis, and cellular senescence. These pathways are most relevant during concentrated, prolonged, repeated, or biologically amplified exposure [3,5,11,12].

### 7.1. ROS Generation, Calcium Dyshomeostasis, and Mitochondrial Feedback

Reactive oxygen species are a recurring signal in local anesthetic cytotoxicity. In neuronal models, bupivacaine-induced apoptosis is associated with reactive oxygen species generation, mitochondrial complex I/III impairment, loss of membrane potential, endoplasmic reticulum stress, and caspase activation. In skeletal muscle, bupivacaine myotoxicity involves oxidative and sarcoplasmic reticulum stress, and N-acetylcysteine attenuates these effects in human skeletal myotubes [57,87].

Calcium dyshomeostasis connects functional toxicity with organelle injury. Local anesthetics may disturb calcium handling through ion channel effects, impaired excitation–contraction coupling, mitochondrial ATP depletion, and ER/SR stress. Increased cytosolic calcium can promote mitochondrial calcium uptake, membrane-potential loss, permeability transition, respiratory chain dysfunction, and further reactive oxygen species generation. ATP depletion then weakens calcium extrusion and reticular reuptake. This creates a self-amplifying sequence linking calcium overload, mitochondrial dysfunction, oxidative stress, and cell death [8,15,28,40].

### 7.2. ER/SR Stress and Apoptotic Signaling

Mitochondrial dysfunction, oxidative stress, and calcium dyshomeostasis can activate endoplasmic/sarcoplasmic reticulum stress. When the unfolded protein response remains adaptive, it may restore proteostasis. When stress is sustained, ER/SR signaling can shift toward apoptosis through PERK/eIF2α/ATF4/CHOP, IRE1-related pathways, Bcl-2 family regulation, cytochrome c release, and caspase activation [52,60,66,87].

Apoptosis is one of the most frequently reported forms of regulated cell death in local anesthetic cytotoxicity. Lidocaine can induce apoptosis through the intrinsic mitochondrial pathway independently of death receptor signaling. Bupivacaine, lidocaine, and ropivacaine have also been associated with mitochondrial apoptotic pathways in neural, skeletal muscle, tendon, and other cellular models. The dominant death route depends on tissue type, concentration, exposure duration, and metabolic state [56,88,89,90].

### 7.3. Autophagy, Mitophagy, and Mitochondrial Quality Control

Autophagy and mitophagy normally protect cells by removing damaged proteins and organelles. They can become harmful when activation is excessive, flux is impaired, or mitochondrial clearance exceeds the cell’s capacity to maintain bioenergetic function. High glucose amplifies bupivacaine cytotoxicity through apoptosis and impaired autophagy, involving PERK–ATF4–CHOP and IRE1–TRAF2 signaling. In dorsal root ganglion neurons, a high-glucose environment also promotes reactive oxygen species-dependent autophagic damage through *TUG1*/mTOR signaling [62,63,66].

Mitophagy illustrates the same double role. Lidocaine neurotoxicity in spinal cord neurons from diabetic Goto-Kakizaki rats involves AMP-activated protein kinase-mediated mitophagy. Ropivacaine-induced neuronal apoptosis is linked to excessive PINK1/Parkin-mediated mitophagy. These findings suggest that mitochondrial quality control can shift from repair to injury when exposure is strong, prolonged, or metabolically amplified [64,65].

### 7.4. Ferroptosis, Ferritinophagy, and Senescence

Ferroptosis has recently been proposed as a pathway in local anesthetic-induced spinal neurotoxicity. This iron-dependent form of regulated cell death involves lipid peroxidation, glutathione depletion, glutathione peroxidase 4 (GPX4) dysfunction, and oxidative membrane injury. In rat models, ferrostatin-1 attenuates bupivacaine-induced neurotoxicity by reducing ferroptosis-related injury. Lidocaine models also suggest ferritinophagy-mediated ferroptosis, with changes in nuclear receptor coactivator 4 (NCOA4), CDGSH iron-sulfur domain-containing protein 2 (CISD2), GPX4, solute carrier family 7 member 11/cystine-glutamate antiporter (SLC7A11/xCT), glutathione, reactive oxygen species, and iron handling. These findings remain experimental and model-specific. They should be presented as plausible injury pathways, not as universal mechanisms of local anesthetic neurotoxicity [68,69].

Local anesthetics may also induce sublethal injury programs. In intervertebral disk cells, lidocaine-induced reactive oxygen species trigger DNA double-strand breaks and cellular senescence through the MYC proto-oncogene (MYC)–dual-specificity phosphatase 1 (DUSP1)–tumor protein p53 (p53) axis. This finding is tissue- and model-specific, but it broadens the toxicity spectrum beyond acute apoptosis or necrosis. In tissues with limited regenerative capacity, persistent cellular dysfunction may be as relevant as immediate cell death [23].

### 7.5. Metabolic and Physiological Vulnerability

Biological context can lower the threshold at which local anesthetic exposure becomes injurious. High-glucose conditions, pregnancy-related experimental vulnerability, developmental state, impaired repair capacity, and reduced mitochondrial reserve all show that toxicity is not determined by concentration and exposure duration alone. These models do not provide direct clinical dose equivalents. They do show that cellular reserve, redox burden, calcium-handling capacity, and repair competence influence whether exposure remains reversible or progresses toward persistent injury [5,11,62,63,66,67,74].

This vulnerability concept is revisited in Section 9.3, where it is considered in relation to pregnancy, pediatric exposure, organ dysfunction, prolonged infusion, and route-specific risk.

### 7.6. Integrated Interpretation

The cellular stress pathways reviewed here explain how local anesthetic toxicity can become self-amplifying. Mitochondrial dysfunction reduces ATP availability and impairs calcium handling. Calcium overload and oxidative stress further injure mitochondria and activate ER/SR stress. If repair and quality control systems fail, apoptosis, ferroptosis-related injury, senescence, or persistent functional impairment may follow. These pathways are most relevant during concentrated, prolonged, repeated, or biologically amplified exposure. They provide the mechanistic transition from systemic functional toxicity to tissue-specific injury [8,12,23,40,52,66,87].

## 8. Organ- and Tissue-Specific Injury

Local anesthetics can produce direct tissue-specific cytotoxicity at or near the site of administration. These effects have been described in several experimental tissue models and are summarized in Table 1. Their relevance depends on route, concentration, duration of exposure, vascular washout, tissue repair capacity, and local vulnerability. Across models, a consistent principle emerges: tissue injury is exposure-dependent and reflects the cellular stress pathways described above [5,11,12,13,91].

A formal quantitative ranking of tissue vulnerability across skeletal muscle, cartilage, peripheral nerve, epithelium, tendon, intervertebral disk, and platelet-related repair systems remains difficult because published studies differ substantially in local anesthetic type, concentration, exposure duration, formulation, washout conditions, cell source, culture platform, and injury readout. Nevertheless, full-text evidence from tissue-focused reviews supports a common concentration–time framework: higher local concentrations, longer exposure, repeated administration, limited washout, reduced regenerative capacity, extracellular-matrix dependence, and reduced mitochondrial reserve increase the likelihood of cytotoxic injury. In cartilage, systematic reviews consistently describe dose- and time-dependent chondrotoxicity, with ropivacaine generally appearing less chondrotoxic at lower clinically used concentrations and corticosteroid coadministration potentially worsening chondrocyte injury in some models. These data support a comparative microenvironmental framework rather than precise universal damage thresholds. Future studies should therefore standardize exposure units, local clearance assumptions, recovery intervals, and tissue-specific endpoints before clinically meaningful tissue damage thresholds can be defined [11,102].

These tissue models do not by themselves show that routine brief, clinically appropriate exposure is unsafe. They show that toxicity signals become most relevant when local concentration, exposure duration, repeated administration, impaired washout, or tissue vulnerability shifts exposure away from brief reversible pharmacology [5,11,13,91,97].

The dominant injury pattern varies by tissue and model. The translational message is more stable: toxicity is shaped by exposure biology, local clearance, tissue reserve, and repair capacity, not by drug identity alone [4,5,11,12,13,21,22,23,87,91,97].

## 9. Translational Implications and Future Directions

Local anesthetic toxicity should not be interpreted only as sodium channel blockade. Sodium channel inhibition remains the main pharmacological action, but the final toxic phenotype depends on exposure biology, tissue vulnerability, mitochondrial and metabolic reserve, membrane effects, and cellular stress responses [1,2,8,9,10,11].

### 9.1. Exposure, Vulnerability, and Injury

A useful translational model should separate exposure from injury. Exposure is shaped by drug chemistry, dose, concentration–time profile, route, tissue distribution, protein binding, clearance, and local washout. Injury develops when this exposure exceeds the reserve of specific cells or tissues. Sodium channel blockade is one layer of this process. Potassium- and calcium-channel effects, receptor-level changes, mitochondrial dysfunction, altered fatty acid metabolism, membrane perturbation, oxidative stress, ER/SR stress, and regulated cell death determine how the phenotype develops [9,23,25,28,31,40,52,68].

This exposure–injury model also explains why the same drug class can produce different clinical and experimental outcomes. Systemic exposure may present as CNS excitation, seizures, myocardial depression, conduction disturbance, or cardiovascular collapse. Local exposure may instead produce myotoxicity, chondrotoxicity, neurotoxicity, epithelial injury, impaired wound healing, or delayed repair. These phenotypes differ clinically, but they share overlapping molecular stress pathways [1,4,6,11,12,21,22].

### 9.2. Implications for Safer Drug Design and Formulations

Small structural and stereochemical differences can change toxicological behavior. Ropivacaine’s lower lipophilicity contributes to its more favorable systemic safety profile. Levobupivacaine is the S-enantiomer of bupivacaine and was developed to reduce the risk associated with racemic bupivacaine, particularly its R-enantiomer-related cardiotoxicity. Lipophilicity and stereochemistry therefore remain important, although their relative influence differs across systemic cardiotoxicity, neurotoxicity, and local tissue injury [2,3,7,18].

Future local anesthetic development should not evaluate sodium channel potency and duration of action alone. Structure–toxicity assessment should also consider mitochondrial liability, membrane partitioning, tissue accumulation, calcium dynamics, oxidative stress, and non-neuronal cytotoxicity. Systemic safety improvements, including S-enantiomeric formulations, may not prevent local tissue injury during high-concentration, repeated, or prolonged exposure [8,10,12,15].

Formulation and route shape exposure biology by creating different concentration–time profiles. Continuous catheters, local infiltration analgesia, intra-articular injections, topical ocular exposure, high-volume fascial plane blocks, and sustained-release preparations expose tissues in different ways. Sustained-release formulations such as liposomal bupivacaine may delay peak plasma concentration and prolong local exposure. These advantages do not remove the need to assess local tissue toxicity, route-specific safety, mitochondrial stress, and repair biology [5,13,22,97,103].

Mixtures of different local anesthetics also require separate consideration. Their combined systemic toxicity may be additive, while the physicochemical compatibility and toxicological behavior of specific mixtures are not always predictable. Combined exposure should therefore be considered rather than treating the maximum dose of each local anesthetic as an independent limit. Direct evidence for specific synergistic mitochondrial or ER/SR toxicity between different local anesthetics remains limited.

Clinically, local anesthetics are also frequently administered with adjuvants such as epinephrine/adrenaline, dexamethasone, and dexmedetomidine. These combinations may modify toxicity-relevant exposure biology by changing local perfusion, systemic absorption, block duration, tissue residence time, inflammatory signaling, neuronal excitability, and motor-block duration. Epinephrine may delay systemic absorption through vasoconstriction, but reduced local blood flow may also be relevant in tissues or patients with pre-existing neural or microvascular vulnerability. Dexamethasone and dexmedetomidine can prolong analgesia, yet available evidence does not establish a universal synergistic mechanism by which these adjuvants consistently amplify mitochondrial oxidative stress, ER/SR stress, or regulated cell death. Preclinical dexamethasone data suggest general safety at low preservative-free doses, but high-dose use, additives, neuropathic conditions, and combination with concentrated local anesthetics remain contexts requiring caution. Future experimental studies should therefore evaluate local anesthetics not only as isolated compounds, but also under clinically relevant combination conditions, with matched concentration–time profiles, route-specific washout assumptions, and tissue-specific endpoints [104,105].

Continuous catheter techniques and prolonged local anesthetic infusions are clinically relevant examples of exposure-dependent risk. They do not imply toxicity by themselves, but they extend the concentration–time profile and may increase cumulative systemic exposure or sustained local tissue contact. Adult truncal catheter data show that toxicity is uncommon, but reported risk contexts include high cumulative 24-h dose, bilateral catheters, prolonged infusion, cardiac surgery, cytochrome P450 (CYP)-inhibiting medications, and hypoalbuminemia [106]. Pediatric truncal catheter data similarly support the importance of age, cumulative dose, prolonged infusion, and toxic blood concentrations, particularly in infants and young children [107]. These data connect pharmacokinetics, cumulative exposure, local clearance, myotoxicity, neurotoxicity, and systemic toxicity risk without turning this review into a clinical guideline [96,108,109].

### 9.3. Biological Vulnerability and Patient-Specific Risk

Biological vulnerability modifies local anesthetic toxicity, but exposure remains the starting point. Free systemic and target-tissue exposure depends not only on total dose, but also on injection site, perfusion, absorption kinetics, concentration–time profile, protein binding, hepatic metabolism, renal reserve, cardiac output, acidosis, pregnancy, developmental stage, concurrent disease, drug interactions, and repeated or continuous administration [24,109]. Continuous catheter literature supports the same principle from a clinical exposure perspective: cumulative dose, high 24-h dose, prolonged infusion, bilateral or multiple catheters, cardiac surgery, CYP-inhibiting medications, hypoalbuminemia, and young age can all modify exposure and risk [106,107,108].

Experimental models add a second layer. Once local anesthetic exposure reaches vulnerable tissue, injury may be amplified by reduced mitochondrial reserve, impaired fatty acid oxidation, disturbed calcium handling, oxidative or ER/SR stress, diabetes-like metabolic stress, pregnancy-related experimental vulnerability, developmental vulnerability, or limited repair capacity [62,66,67,74,77]. These models should not be used as direct clinical dose equivalents. Their value is mechanistic: they identify biological contexts in which toxicity may be amplified through altered exposure, reduced metabolic reserve, impaired mitochondrial function, or limited tissue repair.

Infancy and early childhood are important developmental exposure contexts, but they are not a separate focus of this review. Reported pediatric LAST cases involve infants and young children disproportionately, plausibly reflecting immature clearance of amide local anesthetics and lower α1-acid glycoprotein (AAG) concentrations, which may increase the unbound drug fraction [24,110]. Pediatric truncal catheter research also identifies cumulative dose, prolonged infusion, multiple catheters, breakthrough boluses, hypoalbuminemia, cardiac surgery, hepatic dysfunction, and metabolic disease as exposure-modifying contexts [107].

Methemoglobinemia is a distinct pediatric-relevant toxicity, especially after prilocaine, benzocaine, or excessive lidocaine–prilocaine topical exposure. Unlike sodium channel-mediated LAST, it reflects hemoglobin oxidation and impaired oxygen transport. Infants may be more susceptible because of lower methemoglobin-reducing capacity, greater oxidizability of fetal hemoglobin, and higher transdermal exposure relative to body weight. This example reinforces the broader point: developmental pharmacokinetics, protein binding, hemoglobin redox biology, cumulative exposure, and oxygen delivery reserve can modify the toxic phenotype. It should not be interpreted here as dosing guidance [111,112].

### 9.4. Experimental Models and Mechanistic Readouts

Current evidence comes from whole-animal studies, isolated organs, cultured cells, subcellular fractions, organelle preparations, and biomimetic membranes. Each model answers a different mechanistic question. None fully reproduces clinical toxicity, systemic pharmacokinetics, tissue perfusion, protein binding, metabolism, local washout, or resuscitation physiology. These limitations are important when high-concentration or simplified systems are used to infer tissue risk [8,10,12,21,25,40].

Human induced pluripotent stem cell-derived cardiomyocyte (hiPSC-CM) models are useful for studying human-relevant cardiomyocyte responses to local anesthetics. They can capture aspects of calcium dynamics, contractile behavior, and drug-specific cardiotoxicity that are difficult to assess in simpler systems. They may also help explore interindividual or disease-related susceptibility. Their findings still need to be interpreted together with pharmacokinetic, tissue-level, and in vivo data [16,40].

Future experimental work should better match clinically relevant exposure patterns. Short exposure, prolonged infusion, repeated bolus administration, depot release, poor washout, and high local concentration are not biologically equivalent. Useful platforms may include three-dimensional cartilage models, engineered muscle tissue, nerve-on-a-chip systems, corneal epithelial models, intervertebral disk models, and co-cultures containing glial, immune, endothelial, or stromal components. These approaches may help separate clinically plausible toxicity from mechanistic stress testing [11,12,13,22,23].

### 9.5. Computational, Multi-Omics, and Systems Toxicology Approaches

Computational and systems toxicology approaches may help organize heterogeneous mechanistic data. In silico models can link chemical properties with pharmacokinetic behavior, membrane partitioning, mitochondrial liability, calcium-related effects, oxidative stress, and tissue-specific endpoints. Their value depends on transparent datasets, defined concentration–time inputs, standardized endpoints, and clear applicability domains [3,10,11,17,40].

Multi-omics approaches may further improve mechanistic interpretation of local anesthetic toxicity. Transcriptomics and proteomics could identify coordinated stress-response networks linked to mitochondrial dysfunction, ER/SR stress, apoptosis, ferroptosis, senescence, and impaired repair, whereas metabolomics and lipidomics could map changes in fatty acid oxidation, acylcarnitine handling, glutathione metabolism, iron-dependent lipid peroxidation, membrane remodeling, and redox balance. Single-cell and spatial approaches may be particularly useful for identifying vulnerable subpopulations within mixed tissue models, including Schwann cells, glial cells, chondrocytes, keratinocytes, tenocytes, and developing oligodendrocyte-lineage cells. Local-anesthetic-specific metabolomics already illustrates this value: in SH-SY5Y neuronal cells, articaine and lidocaine produced metabolic changes involving glycolysis, glucose-dependent pathways, branched-chain amino acid catabolism, tricarboxylic acid (TCA) cycle fueling, choline metabolism, and lipid droplet handling, whereas Schwann cell studies combining extracellular metabolic flux analysis and nuclear magnetic resonance (NMR) metabolomics identified distinct toxic pathways after articaine and lidocaine exposure, including oxygen-consumption impairment, ER-stress signaling, and apoptosis-related markers. Integrated with pharmacokinetic/pharmacodynamic (PK/PD) modeling and exposure modeling, these approaches could help distinguish adaptive stress responses from irreversible molecular injury and identify biomarkers of tissue-specific vulnerability [21,113,114].

Predictive models should not rely only on chemical descriptors. Local anesthetic toxicity depends on both molecular structure and biological context. Computational frameworks therefore need to incorporate exposure biology, route-specific concentration–time profiles, tissue vulnerability, mitochondrial reserve, and repair capacity [5,11,16,17].

### 9.6. Integrated Interpretation

**Figure 3:**
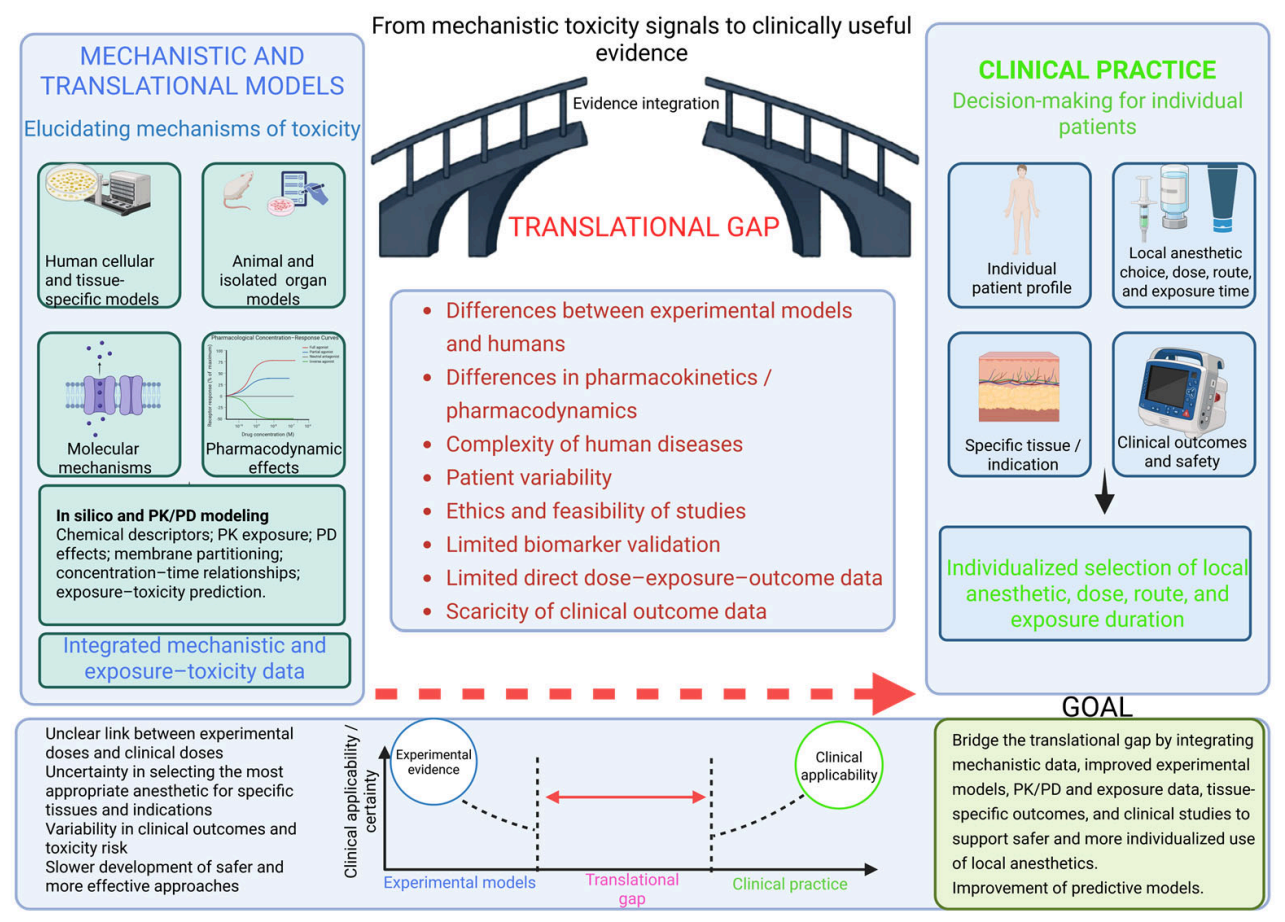
From mechanistic toxicity signals to clinically useful evidence. Arrows indicate the proposed translational flow: the large red arrow shows progression from mechanistic and experimental evidence toward clinical practice; the bidirectional red arrow in the lower inset depicts the translational gap between experimental models and clinical applicability; and the black arrow in the clinical-practice panel links patient, tissue, treatment, and outcome factors to individualized local anesthetic selection. Created in BioRender. Rijavec, B. (2026) https://BioRender.com/frr97fi (accessed on 28 July 2026).

Future safety assessment should extend beyond sodium channel pharmacology. It should combine chemical structure, concentration–time exposure, tissue susceptibility, mitochondrial and metabolic reserve, and repair capacity. Mixture-specific assessment is also required because local anesthetic–local anesthetic and local anesthetic–adjuvant combinations may show additive systemic toxicity, physicochemical incompatibility, precipitation or crystallization, while their clinical benefit is not consistently demonstrated; direct evidence for synergistic mitochondrial oxidative stress or ER/SR injury remains limited [115]. Where feasible, these assessments should be linked to multi-omics, single-cell, and tissue-specific readouts rather than relying only on sodium channel potency or bulk viability endpoints. Such models may better distinguish reversible pharmacological effects from molecular injury and may improve route-specific assessment of systemic and local tissue risk [1,2,3,8,16,17,40]. Figure 3 summarizes this translational gap and illustrates how mechanistic toxicity data may be integrated with exposure biology, tissue-specific outcomes, and clinical evidence.

Mechanistic and translational models, including human cellular and tissue-specific models, animal and organ models, molecular mechanisms, pharmacodynamic effects, and in silico pharmacokinetic/pharmacodynamic modeling, help connect toxicity mechanisms with exposure–effect relationships, toxic potency, and route-specific risk. Species differences, pharmacokinetic variability, patient heterogeneity, disease complexity, ethical constraints, limited biomarker validation, and scarcity of direct clinical outcome data create a translational gap. Bridging this gap requires integration of mechanistic data, improved experimental models, PK/PD and exposure data, tissue-specific outcomes, and clinical studies to support safer individualized selection of local anesthetic type, dose, route, and exposure duration. The intended outcome is improved predictive modeling for safer route-specific and individualized local anesthetic selection.

## 10. Conclusions

Local anesthetic toxicity cannot be explained by voltage-gated sodium channel blockade alone. Sodium channel inhibition remains central to therapeutic action and early toxicity, but the final injury pattern also depends on membrane effects, receptor and ion channel dysregulation, mitochondrial and metabolic dysfunction, calcium dyshomeostasis, oxidative stress, ER/SR stress, impaired quality control pathways, and regulated cell death [1,2,8,9,10,11,52,66].

Bupivacaine remains the main example of disproportionate cardiotoxicity because high lipophilicity, persistent cardiac ion channel effects, myocardial accumulation, mitochondrial impairment, altered fatty acid handling, and membrane-level injury occur together. Ropivacaine and levobupivacaine have a more favorable systemic safety profile than racemic bupivacaine, but this does not exclude mitochondrial, cellular, or tissue-specific toxicity during high-concentration, repeated, or prolonged exposure [2,3,7,8,9,10,15,79,82,83,84].

Lidocaine illustrates a different toxicity pattern. It is less associated with disproportionate systemic cardiotoxicity than bupivacaine, but it remains important in local and neural toxicity models. High-concentration or neuraxial exposure has been linked to mitochondrial injury, caspase activation, ER stress-associated apoptosis, spinal neurotoxicity, and sublethal tissue injury such as intervertebral disk cell senescence. This distinction is important: a more favorable systemic cardiac profile does not exclude concentration- and exposure-dependent cellular injury at the site of administration [23,52,56,60,70].

Direct tissue-specific toxicity extends local anesthetic injury beyond systemic toxicity. Localized injury differs between skeletal muscle, cartilage, peripheral nerve, spinal tissue, epithelial surfaces, intervertebral disk cells, tendon cells, platelet-related repair environments, and developing neural cells, but several mechanisms recur across models. These findings should not be read as direct clinical dose equivalents. They show that local toxicity depends on route, concentration, exposure duration, tissue perfusion, vascular clearance, and repair capacity [4,5,11,12,21,22,23,87].

Prolonged exposure patterns deserve particular attention. Continuous catheter techniques, repeated administration, high-volume local techniques, and sustained-release formulations can separate systemic plasma kinetics from sustained local tissue contact. They do not imply toxicity by themselves, but they make concentration–time exposure, cumulative dose, washout, and tissue vulnerability more important than in single-dose models [103,106,107,108].

Future studies should link local anesthetic type, concentration, formulation, route, exposure duration, patient vulnerability, and tissue-specific outcomes. Without exposure-linked data, mechanistic toxicity signals cannot be translated reliably into route-specific safety assessment or tissue-specific drug selection [4,5,11,12,13,16,17,21,22,23,87].

## Figures and Tables

**Table 1:** Tissue-specific patterns of local anesthetic toxicity.

Tissue/Model	Exposure Context	Reported Mechanisms in Selected Models	Key References
Skeletal muscle/myotubes	Surgical wound infiltration, repeated or prolonged exposure, continuous regional analgesia, and surgical wound models	Mitochondrial dysfunction, oxidative stress, sarcoplasmic reticulum stress, calcium dyshomeostasis, ATP depletion, altered myogenic or regeneration markers, delayed recovery, impaired repair responses, and myotoxicity in experimental models	[87,92,93,94,95,96]
Cartilage/chondrocytes	Intra-articular exposure, repeated or prolonged local exposure	Reduced chondrocyte viability, mitochondrial dysfunction, mitochondrial membrane potential loss, mitochondrial ROS generation, apoptosis or caspase activation, impaired proliferation, and autophagy-related changes	[12,13,91,97,98]
Peripheral nerve/Schwann cells and neuronal cell models	Perineural or intraneural exposure, high local concentration, dental or peripheral nerve-related models, and repeated or prolonged exposure	Time- and concentration-dependent Schwann cell injury, mitochondrial bioenergetic impairment, metabolic remodeling, ER stress signaling, apoptosis-related markers, and concentration-dependent neuronal cytotoxicity.	[21,99,100]
Spinal cord/neuraxial experimental models	Neuraxial exposure, high local concentration, repeated or prolonged exposure, and intrathecal or epidural experimental models	Direct spinal neural injury, concentration-dependent histopathological and ultrastructural damage, mitochondrial dysfunction, calcium dyshomeostasis, inflammatory signaling, and emerging autophagy/mitophagy or ferroptosis/ferritinophagy pathways.	[68,69,70,71,72]
Keratinocytes/cutaneous wound healing models	Ropivacaine exposure in rat wound models and HaCaT human keratinocyte cells	Delayed wound closure, reduced epidermal thickness, suppressed keratinocyte proliferation and migration, increased apoptosis, phosphoinositide 3-kinase (PI3K)/Akt/mTOR suppression, and altered interleukin 6 (IL-6), tumor necrosis factor alpha (TNF-α), and interleukin 10 (IL-10) responses	[4]
Corneal epithelium/ocular surface	Short topical ocular exposure in 2D/3D corneal epithelial models and ex vivo porcine cornea	Drug-dependent epithelial cytotoxicity; oxybuprocaine showed the strongest reduction in viability and delayed wound healing, whereas lidocaine and bupivacaine were less cytotoxic and did not impair wound healing in the porcine model	[22]
Intervertebral disk cells	Lidocaine exposure in nucleus pulposus cells and mouse intervertebral disk degeneration model	ROS generation, DNA double-strand breaks, DNA damage response, MYC–DUSP1–p53 signaling, cellular senescence, and extracellular matrix catabolic changes	[23]
Rotator cuff tendon cells/tenocytes	Lidocaine exposure in human and rat rotator cuff tenocytes	Reduced viability, apoptosis, reduced Bcl-2 expression, cytochrome c-related mitochondrial apoptotic signaling; neurotropin attenuated these effects in vitro	[89]
Platelet-related repair environments	Exposure of platelet preparations to lidocaine, ropivacaine, or bupivacaine in platelet-rich-plasma-related conditions	Bupivacaine produced platelet shrinkage, calcium dysregulation, ROS generation, apoptosis, reduced viability, and impaired adhesion; lidocaine and ropivacaine showed smaller or limited effects in the same model	[101]
Developmental spinal cord/oligodendrocyte precursor cells (OPCs)	Experimental neonatal intrathecal ropivacaine exposure and primary OPC models	Developmental dysmyelination, sensory dysfunction, impaired OPC maturation, reduced myelin basic protein (MBP) expression, Akt suppression, attenuated Ca^2+^ oscillations, mitochondrial dysfunction, and increased ROS	[74]

## Data Availability

No new data were created or analyzed in this study. Data sharing is not applicable to this article.

## Abbreviations

The following abbreviations are used in this manuscript:

AAG	α1-acid glycoprotein
Akt	protein kinase B
AMPK	AMP-activated protein kinase
ATP	adenosine triphosphate
ATF4	activating transcription factor 4
ATF6	activating transcription factor 6
Bak	Bcl-2 homologous antagonist/killer
Bax	Bcl-2-associated X protein
Bcl-2	B-cell lymphoma 2
Bcl-xL	B-cell lymphoma-extra large
BDNF	brain-derived neurotrophic factor
BiP	binding immunoglobulin protein
Ca^2+^	calcium ion
CaV1.3	voltage-gated calcium channel subtype 1.3
CHOP	C/EBP homologous protein
CISD2	CDGSH iron-sulfur domain-containing protein 2
CNS	central nervous system
CYP	cytochrome P450
DRG	dorsal root ganglion
DRP1	dynamin-related protein 1
DUSP1	dual-specificity phosphatase 1
eIF2α	eukaryotic initiation factor 2 alpha
ER	endoplasmic reticulum
ER/SR	endoplasmic/sarcoplasmic reticulum
GABA	γ-aminobutyric acid
GABA-A receptor	γ-aminobutyric acid type A receptor
GPX4	glutathione peroxidase 4
GSK-3β	glycogen synthase kinase-3 beta
HaCaT	human keratinocyte cell line
HCN	hyperpolarization-activated cyclic nucleotide-gated channel
hiPSC-CM	human induced pluripotent stem cell-derived cardiomyocyte
Ih	hyperpolarization-activated current
IL-6	interleukin 6
IL-10	interleukin 10
IRE1	inositol-requiring enzyme 1
KATP	adenosine triphosphate-sensitive potassium channel
Kv3	voltage-gated potassium channel 3
LAST	local anesthetic systemic toxicity
lncRNA	long non-coding RNA
M2 receptor	muscarinic acetylcholine receptor subtype 2
MBP	myelin basic protein
METTL3	methyltransferase-like 3
mRNA	messenger RNA
mTOR	mechanistic target of rapamycin
MYC	MYC proto-oncogene
Na^+^/K^+^-ATPase	sodium–potassium adenosine triphosphatase
NaV	voltage-gated sodium channel
NaV1.5	cardiac voltage-gated sodium channel isoform 1.5
NCOA4	nuclear receptor coactivator 4
NMDA receptor	N-methyl-D-aspartate receptor
NMR	nuclear magnetic resonance
OPC	oligodendrocyte precursor cell
p53	tumor protein p53
PARP-1	poly(ADP-ribose) polymerase 1
PC12	rat pheochromocytoma cell line
PERK	protein kinase R-like endoplasmic reticulum kinase
PI3K	phosphoinositide 3-kinase
PINK1	PTEN-induced kinase 1
PK/PD	pharmacokinetic/pharmacodynamic
pKa	acid dissociation constant
PR interval	electrocardiographic interval from atrial depolarization to ventricular depolarization
PRISMA	Preferred Reporting Items for Systematic Reviews and Meta-Analyses
QRS complex	electrocardiographic marker of ventricular depolarization
QT interval	electrocardiographic interval from ventricular depolarization to repolarization
ROS	reactive oxygen species
SH-SY5Y	human neuroblastoma cell line
SK2	small-conductance calcium-activated potassium channel type 2
SLC7A11	solute carrier family 7 member 11
SR	sarcoplasmic reticulum
TASK	TWIK-related acid-sensitive potassium channel
TCA	tricarboxylic acid
TNF-α	tumor necrosis factor alpha
TRAF2	tumor necrosis factor receptor-associated factor 2
TREK-1	TWIK-related potassium channel 1
*TUG1*	taurine-upregulated gene 1
XBP1	X-box binding protein 1
xCT	cystine/glutamate antiporter
